# FSH prevents depletion of the resting follicle pool by promoting follicular number and morphology in fresh and cryopreserved primate ovarian tissues following xenografting

**DOI:** 10.1186/1477-7827-10-98

**Published:** 2012-11-24

**Authors:** Viktoria von Schönfeldt, Ramesh Chandolia, Robert Ochsenkühn, Eberhard Nieschlag, Ludwig Kiesel, Barbara Sonntag

**Affiliations:** 1 Department of Obstetrics and Gynecology, Campus Grosshadern Ludwig-Maximilians-University Munich, Munich, Germany; 2 Center for Reproductive Medicine and Andrology, University of Münster, Münster, Germany; 3 Center for Reproductive Medicine, Munich, Germany; 4 Department of Gynecology and Obstetrics, University of Münster, Münster, Germany; 5 Zentrum für Endokrinologie, Kinderwunsch und Pränatale Medizin, MVZ amedes, Hamburg, Germany

**Keywords:** Ovary, Cryopreservation, Xenografting, Fertility preservation, FSH

## Abstract

**Background:**

Cryopreservation and transplantation of ovarian tissue is one option for re-establishing ovarian function, but optimal conditions for graft sustainment and follicular survival are still considered experimental. The present study aims to analyze the effect of FSH treatment on the resting follicle pool in fresh and cryopreserved primate ovarian tissues following xenografting.

**Methods:**

Ovarian tissues from adult marmosets were grafted freshly or following cryopreservation to ovarectomized nude mice treated with FSH 25 IU twice daily post transplantation or left untreated as controls. Grafts were retrieved 2 or 4 weeks after transplantation to evaluate the number and morphological appearance of follicles.

**Results:**

Early start of FSH treatment within 1 week following transplantation partly prevents primordial follicle loss in fresh and frozen-thawed tissues, whereas after a 3 weeks time interval this effect is present only in fresh tissues. A similar positive effect of early, but not later FSH treatment on primary follicles is seen in fresh tissues compared to only marginal effects in frozen-thawed tissues. The percentage of morphologically normal follicles is generally increased in FSH treated tissues, whereas the percentage of primary follicles over all primordial and primary follicles is increased by FSH only in freshly-grafted tissues.

**Conclusions:**

FSH treatment alleviates depletion of the resting follicle pool and promotes normal follicular morphology both in freshly and frozen-thawed grafted tissues. In previously cryopreserved tissues, applying to most of the tissues intended for clinical use in fertility preservation attempts, its positive effect on primordial follicle numbers and potential graft sustainment is dependent on an early start of treatment within one week of transplantation.

## Background

Increasing cancer survival rates and awareness of fertility as a quality of life issue in long-term cancer survivors have fuelled the growing demand for fertility preservation in recent years [1]. In females, cryopreservation of ovarian tissue is one option to preserve fertility from the potentially harmful iatrogenic effects of chemotherapy or irradiation: it bears the advantage of a large follicular number to be stored for future re-establishment of ovarian function both in reproductive and endocrinological terms [2]. Successful application in human cancer survivors has been documented by more than ten reported live births so far. Furthermore, potential long-term survival of grafted tissue has been documented [3,4]. Despite these promising results from a few centers with expertise, conditions of tissue preparation, storage and usage still require further research.

Xenografting has primarily been used as a tool to study follicular development and exclude potential reintroduction of malignant cells by homologous retransplantation. Mostly laboratory and domestic animal models have been employed [5], but in some aspects, e.g. follicular recruitment and growth, the non-human primate model may offer better comparability. The common marmoset has served as a model for human reproduction because of its 28-day ovarian cycle, multiple, non-seasonal ovulations in each cycle, and small size as well as high fecundity in captivity. Recently, refinement of ovarian stimulation to compensate for the relative insensitivity to human gonadotrophins allowed retrieving large numbers of mature and developmentally competent oocytes [6]. Although the marmoset requires unusually high doses of FSH and hCG for superovulation compared with other non-human primates or humans, a recent *in vitro* study has confirmed equal effectiveness of human recombinant FSH compared to marmoset FSH on the marmoset FSH receptor [7]. Therefore, the marmoset as a non-human primate model can further promote success in cryopreservation of human ovary tissues for fertility preservation, as the accessibility of human ovarian tissue for experimental studies is highly limited and data are physiologically closer to humans than those retrieved in the more common murine models.

We have previously evaluated the effect of cryopreservation on marmoset ovarian tissue using a xenograft model [8], demonstrating resumption of follicular growth and sustained graft viability [9]. As was previously demonstrated in other species, xenografting significantly challenged follicular numbers and morphology in that study. Additionally, the effect of ovarectomy on the ratio of primary to primordial follicles had lead us to speculate about the role of gonadotrophins on the resting follicle pool as the major determinant of extended graft survival. Although most human live births following ovarian tissue cryopreservation and retransplantation so far have resulted from spontaneous conception [10], FSH treatment for mild stimulation [11] or IVF treatment [12] may be additionally employed. FSH is the major regulator of advanced follicular growth depending on the presence of FSH receptor from the antral follicle stage on. As follicles do not have functional FSH receptors until the secondary stage, FSH is unlikely to exert direct actions on primordial or primary follicles, because functional gonadotrophin receptors have not yet developed in them. However, indirect effects of FSH on the non-growing follicle pool involving intraovarian regulatory mechanisms, stromal or endothelial factors, and those being produced during advanced follicular growth have been hypothesized upon [13]. In this context, the possible benefits of FSH treatment for enhanced grafting conditions or antral follicle development must be balanced against presumable effects to follicular recruitment decisive for long-term survival of the graft. The present study aims to analyze the effect of FSH treatment on the resting follicle pool in fresh and cryopreserved primate ovarian tissues following xenografting.

## Methods

### Animals

All handling and experimental procedures concerning the animals utilized in this study were in accordance with the German animal protection law (license no. AZ 50.0835.1.0).

Ovarian tissues were taken from 10 healthy female common marmosets with proven fertility aged between 2 and 3 years originating from the breeding colony of our institute’s animal research unit. Handling of animals and tissues was grossly as described previously [8], with some modifications owing to the study purpose as detailed below. Directly upon removal, ovaries were rinsed briefly in phosphate buffered saline (PBS, Gibco, Paisley, UK), transferred into fresh PBS supplemented with human serum albumin (HSA, 4 mg/mL, Irvine Scienfific, Irvine, USA) and dissected under microscopic control at room temperature. Fragment sizes of 1–2 mm^3^ were used in all experiments. Control tissues were fixed immediately; remaining tissues were either grafted freshly or assigned to cryopreservation with dimethylsulfoxide (DMSO).

### Cryopreservation

Cryopreservation was performed with PBS containing DMSO (1.5 mol/L, Merck, Darmstadt, Germany), sucrose (0.5 mol/L, Sigma Aldrich) and HSA (10 mg/mL). Fragments were gently agitated at room temperature (RT) for 20 minutes (min), transferred into cryostraws (Consarctic, Schoellkrippen, Germany) with 0.5 mL of cryoprotectant solution, sealed and cryopreserved following a slow-freezing protocol [14] in a controlled rate programmable freezing system (Consarctic): starting at RT, the cooling rate to – 7°C was 2°C/min. Seeding was induced manually at −7°C. Cooling then continued at 0.3°C/min to −40°C followed by −10°C/min to −150°C. Cryostraws were subsequently immersed in liquid nitrogen and stored for a minimum time period of 2 weeks.

On the day of transplantation, ovarian tissue fragments were thawed following a rapid procedure: straws were warmed at room temperature for 40 seconds and then immersed in a water bath at 30°C until all ice crystals vanished. The contents of the straws were then released into cryoprotectant solution. The cryoprotectant was removed by washing the tissue fragments in the same solutions used for cryopreservation in reversed order with decreasing concentrations (75, 50 and 25% of the original concentration) at RT for 5 min each followed by PBS (with 10 mg/mL HSA) and either grafted as quickly as possible or fixed for histological evaluation (see below).

### Surgical procedures

32 Nu/Nu nude mice (Crl:NU-FoxnI^nu^) were obtained (Charles River Germany, Sulzfeld, Germany) and housed in the central animal facility of the university for at least one week prior to procedures. The environment was air filtered with a 12-hour light: 12-hour dark cycle at 23°C; all animals had free access to sterilized food and water. Ovarectomies were carried out two weeks prior to grafting through a dorsomedian incision; surgical procedures were performed under a laminar flow hood in aseptic conditions. The animals were anesthetized with 0.15 mg/g body weight ketamine and 2% (v/v) xylazine intraperitoneally (IP). They were kept on a warming plate; eye protection was carried out with humidified sterile swabs. On the day of transplantation, mice received 6–8 grafts per animal. For best accessibility and to achieve a maximum number of grafts per host animal the tissue was placed in subcutaneous pouches formed with a blunt probe in a small incision in the dorsal skin. Each graft was placed in an individual subcutaneous pouch. The grafting experiments have been carried out in multiple rounds to ensure optimal technical handling and minimize the number of animals to be sacrificed. Grafting procedure was scheduled to the availability of freshly donated ovarian tissue. For cryopreserved tissues, storage time was a minimum of 4 weeks, and could also be longer without effect on later analysis To avoid bias inflicted by different endogenous hormonal milieus of the recipients, cryopreserved fragments from both treatment groups along with fresh control tissues were transplanted to every mouse except in the first round of experiments, when only fresh tissue was grafted. The grafting pattern (3–4 grafts were placed on either side of the spinal column) was recorded in all animals for easy identification of the grafts at the end of the experiment. Pouches were sutured with 5/0 vicryl sutures. No animal died during surgery or was lost prior to the end of the experiment.

### FSH treatment of xenografted ovarian tissue

**Figure 1:**
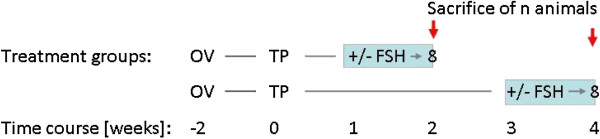
**Experimental design.** Recipient mice were ovarectomized (OV) bilaterally 2 weeks prior to transplantation (TP) of fresh or frozen adult marmoset ovarian tissue from various donor animals as detailed in Table 1. Mice assigned to the treatment groups received a twice daily i.m injection of recombinant human Follitropin beta (25 IU) for 6 days starting 1 week prior to sacrifice; control animals were left untreated. All animals were sacrificed for graft retrieval as indicated (red arrows) either 2 (8 animals in each group) or 4 weeks (8 animals in each group) post transplantation.

In the treatment group, 16 mice bearing an equal number of fresh and frozen grafts were stimulated with 25 IU of FSH i.m. (Gonal-F, MerckSerono, Darmstadt, Germany), twice daily [15] for 6 consecutive days beginning either one week (8 animals) or 3 weeks (8 animals) after transplantation of the grafts. Timing of FSH treatment and explantation relative to previous grafting procedure is detailed in Figure 1.

**Table 1:** Assignment of adult marmoset ovarian tissue fragments to experimental groups

**No**. **of donor animals (n)**	**Experimental group**	**Overall no. of tissue fragments per donor/ total**	**Total no. of analysed follicles**	
10	Freshly fixed	5/50		
			**Untreated**	**FSH**
	Freshly grafted	10/100	4590	9501
	Frozen grafted	10/100	5522	9323

### Graft retrieval and procession of tissue

On the day of graft explantation, the mice were sacrificed by cervical dislocation. Grafts were retrieved either 2 or 4 weeks after the transplantation procedure (Figure 1) and immediately fixed in 4% paraformaldehyde (Sigma Aldrich). Following fixation, tissues were dehydrated in a graded ethanol series, clarified with xylene and routinely embedded in paraffin wax. Serial sections of 5 μm thickness were cut; every fifth section was stained with haematoxylin-eosin (HE) and analysed with a light microscope (Axiophot, Zeiss, Oberkochen, Germany) under a 400 fold magnification. The thickness of tissue sections was determined based on follicle sizes and previous work with marmoset and primate ovarian tissue [16,17]. Marmoset primordial follicles have a diameter of 30 μm, increasing up to 45–85 μm in primary follicles [18]. On the opposite end, small follicles with a nucleus < 19 μm are considered non-growing in primates [19]. Given these numbers, the aforementioned tissue section thickness is adequately chosen to detect the large majority of follicles while at the same time prevent double-counting of follicles. An additional oocyte staining technique has therefore not been employed.

### Histological evaluation

All sections were analyzed by a single investigator blinded to the experimental groups. In the analyzed sections, all follicles were counted. To avoid over counting, follicles were only counted when the nucleus of the oocyte was observed in the section. The developmental stages of the follicles were classified according to Gougeon [20]: the oocyte of a primordial follicle is surrounded by a single layer of flattened granulosa cells (GCs) or a mixture of flattened and cuboidal GCs. In a primary follicle, the single GC layer is cuboidal; a secondary follicle displays two or more layers of GCs; in a preantral follicle small fluid-filled cavities are observed between the GCs and in the antral follicle an antrum is distinguishable.

Follicular morphology was evaluated as described previously [8] based on variables such as integrity of the oocyte, the GCs and the basement membrane. Follicles were classified as morphologically normal if they showed no pyknosis of the oocyte nucleus, no disorganized or detached GCs or shrunken ooplasm.

In general, data are presented as mean numbers of follicles per section for each subclass (primordial and primary follicles) of the resting follicle pool. Additional analyses have been included where appropriate to stress more subtle functional aspects concerning follicular morphology (presented as percentage of morphologically normal follicles over all follicles counted) and percentage of primary follicles calculated over all un-advanced follicles (primordial and primary) reflecting activation process in the resting follicle pool.

### Statistical analysis

Comparisons between two experimental groups were carried out using student´s *t*-*test*, and in case of more than two experimental groups ANOVA was performed with Tamahane for posthoc comparisons in case of statistical significance. Values were considered statistically significant with P < 0.05 and are presented as mean +/− SE. Data analysis was carried out utilizing SPSS for Windows (Version 15.0). Different letters upon bars indicate statistical significance in all graphs.

## Results

### Histological analysis of xenotransplanted frozen ovarian tissues

**Figure 2:**
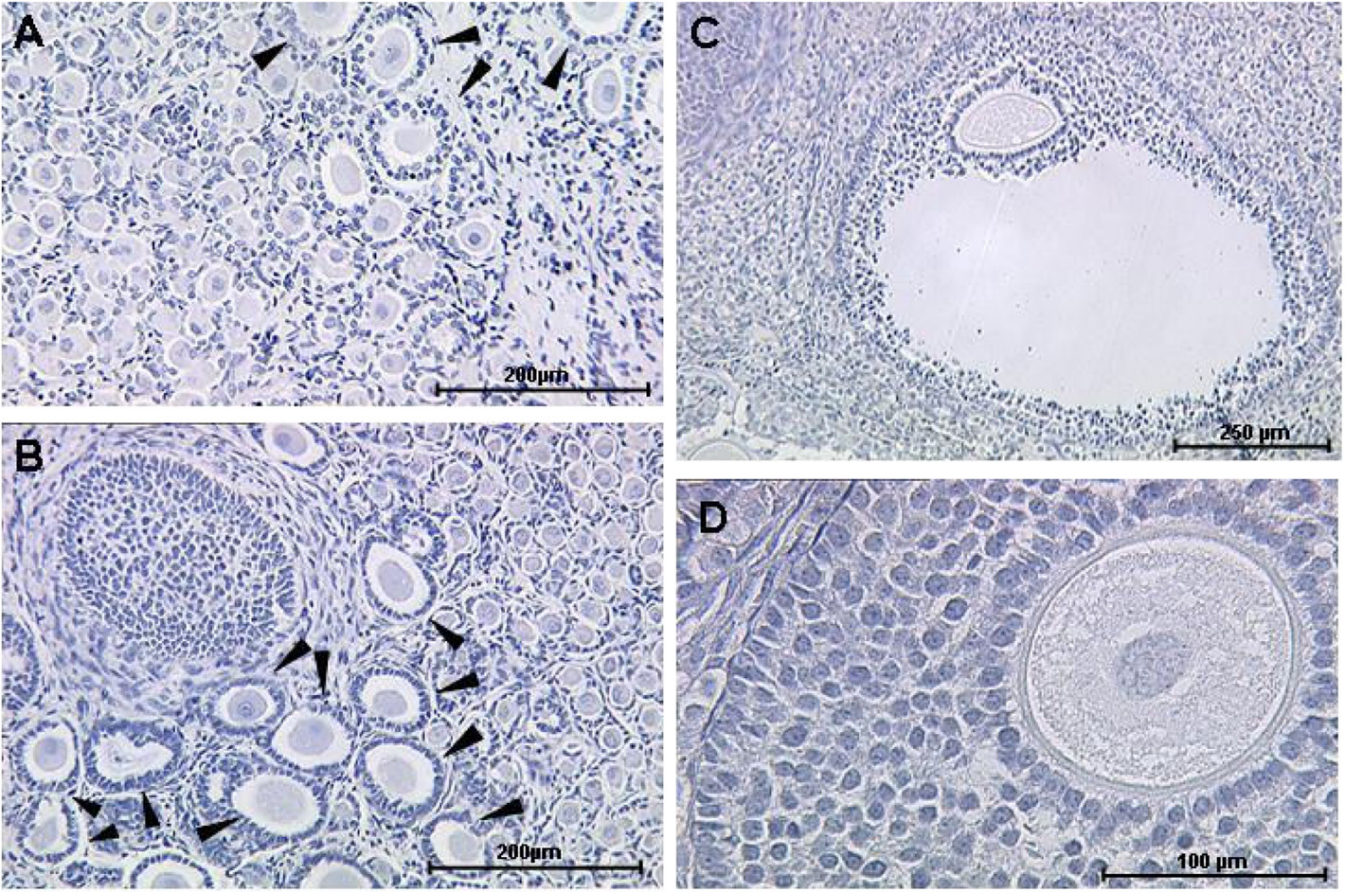
**Typical morphology of frozen**-**thawed ovarian tissues following grafting with or without FSH.** After a grafting period of 4 weeks, cryopreserved adult marmoset ovarian tissues treated with FSH (**B**, overview; **C, D**) displayed all types of follicles including antral (**C**) with largely intact morphology (**D**). Density of follicles advanced past primary stage (**B**, arrowheads) appeared higher than in unstimulated tissues (**A**, overview), but was not evaluated systematically due to low total number of tissue sections with advanced follicles.

Following 2 and 4 weeks after xenotransplantation a total of 127 grafts were retrieved amounting to a graft retrieval rate of 63.5% not differing significantly between experimental groups. The total number of analysed follicles in the different treatment groups is given in Table 1. Examples for the morphology of untreated (Figure 2A) frozen-grafted tissue and frozen-grafted tissue treated with FSH (Figure 2B) are shown in Figure 2. Even though cryopreservation and initial ischemia post grafting severely challenges follicular morphology, advanced follicles found in grafted tissues and the oocytes within displayed a grossly intact morphology (Figure 2D). However, the total number of secondary, pre-antral and antral follicles (Figure 2C) remained insufficiently low to perform statistical analysis in all treatment groups.

Xenografting of ovarian tissue leads to a dramatic initial loss of follicles compared to the pre-graft status of the tissue, which is even more pronounced if the tissue is transplanted following cryopreservation: the mean number of un-advanced (primordial and primary) follicles per section of untreated cryopreserved ovarian tissue is significantly reduced from 45.3+/−5.1 (pregraft) to 6.8+/−0.7 (p=0.001) at 2 weeks after transplantation, and to 7.6+/−2.2 (p=0.001) at 4 weeks after transplantation.

### FSH effects on follicular loss in fresh and frozen-thawed tissues following xenotransplantation

**Figure 3:**
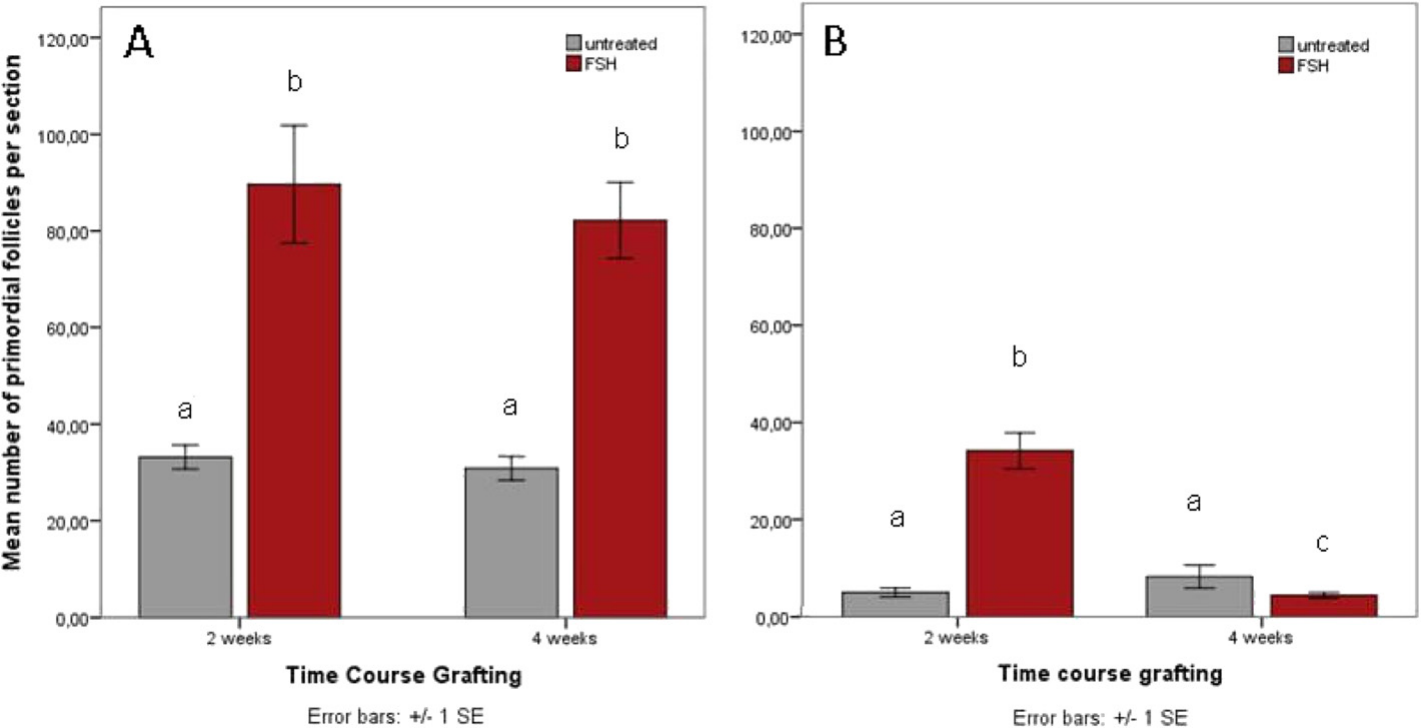
**A and B Effect of FSH treatment on primordial follicle numbers.** FSH-treatment prevents loss of primordial follicles in fresh tissues (**A**) at 2 and 4 weeks post grafting. In frozen tissues (**B**) this effect is present at 2 weeks but reversed at 4 weeks post grafting.

Irrespective of FSH treatment, the overall number of un-advanced follicles is substantially higher in freshly grafted tissues compared to tissues grafted after cryopreservation [mean number of un-advanced follicles per section after grafting for 2 weeks: 37.5+/−2.5 (fresh) vs 8.2+/−0.9 (frozen), p=0.000; mean number of un-advanced follicles per section after grafting for 4 weeks: 36.0+/−3.1 (fresh) vs 9.3+/−2.4 (frozen), p=0.000]. Grafting duration has no influence on mean primordial follicle numbers (Figure 3A): these remain unchanged from 2 weeks (33.2+/−2.5 follicles per section) to 4 weeks (30.8+/−2.5; p = 0.5) after transplantation for untreated tissues.

Treatment with FSH for 6 days prior to explantation rescues follicle numbers to a certain extent compared to untreated tissues: in freshly grafted tissues, FSH application results in a significantly higher mean primordial follicle number per section in treated (81.3+/−10.2, at 2 weeks after transplantation) compared to untreated tissues (33.2+/−2.5 at 2 weeks after transplantation, p=0.001; Figure 3A). This protective effect remains unchanged even when postponing the start of FSH application to 3 weeks after grafting (Figure 3A, 4 wks; p=0.000). Grafting duration, however, did not influence mean primordial follicle number in FSH treated tissues.

In frozen-grafted tissues, mean primordial follicle numbers were likewise positively effected by FSH treatment (34.2+/−3.7, FSH vs 4.9+/−0.9, untreated; p=0.000; Figure 3B), but only if stimulation started shortly after transplantation (explantation at 2 weeks post grafting). At 4 weeks post grafting, mean primordial follicle numbers were significantly lower in the FSH group (4.3+/−0.6, FSH vs 8.2+/−2.4, untreated; p=0.019), whereas mean primordial follicle number did not change over time in the untreated group (p=0.472, Figure 3B).

**Figure 4:**
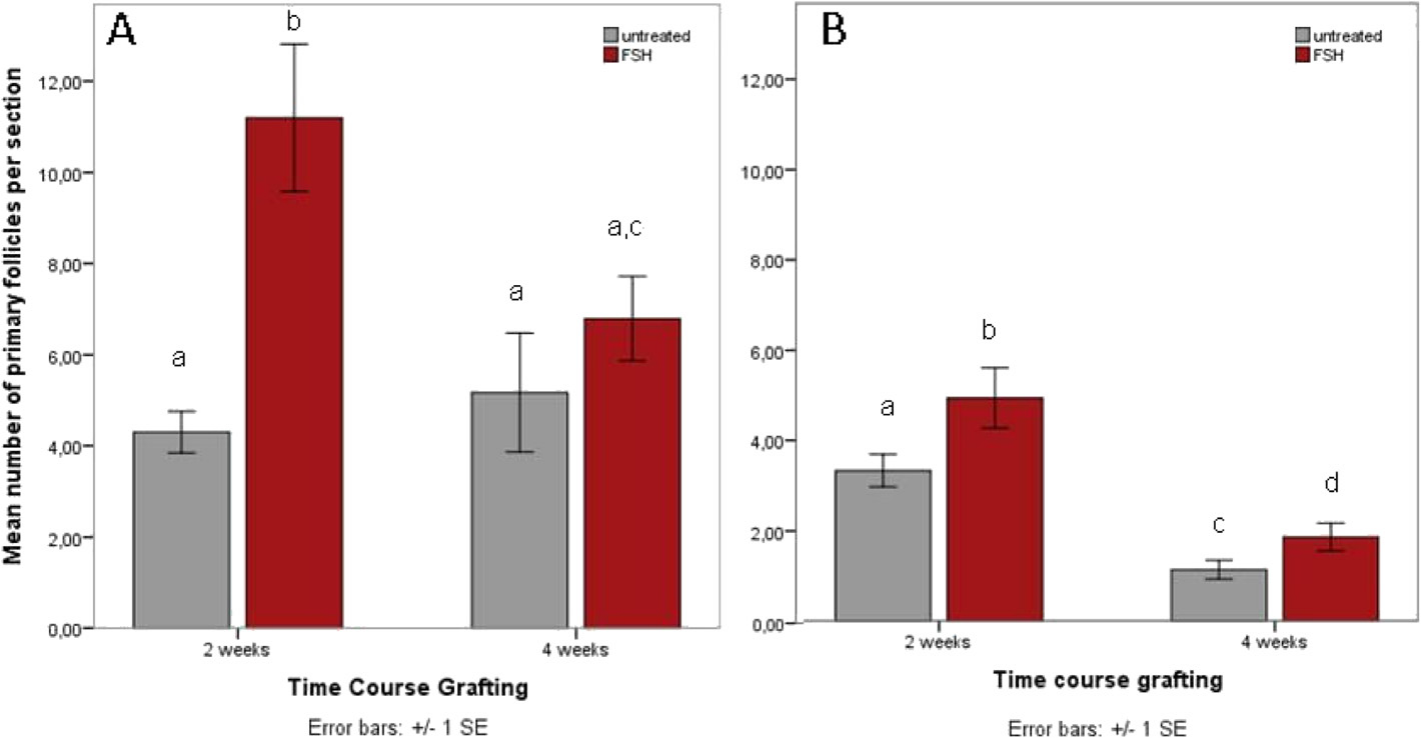
**A and B Effect of FSH treatment on primary follicle numbers.** FSH treatment results in significantly higher mean numbers of primary follicles in fresh ovarian tissues (**A**) at 2 weeks, but not 4 weeks postgraft, with no effect of grafting duration on mean primary follicle number. In frozen tissues (**B**), the pool of primary follicles is decreased over time in both FSH treated and untreated tissues, and FSH treatment significantly increases the mean number of primary follicles 2 weeks and 4 weeks after grafting.

For primary follicles, a positive effect of FSH treatment on mean number is present in freshly grafted tissues at 2 weeks (p=0.008) but not at 4 weeks (p=0.131; Figure 4A) post grafting. In frozen-grafted tissues, mean primary follicle numbers are significantly higher in FSH treated tissues at 2 weeks (p=0.02) and at 4 weeks (p=0.010; Figure 4B) post grafting. The pool of primary follicles is decreased over time in both groups (untreated and FSH, p<0.05; Figure 4B).

### FSH promotes follicular morphology following xenotransplantation

**Figure 5:**
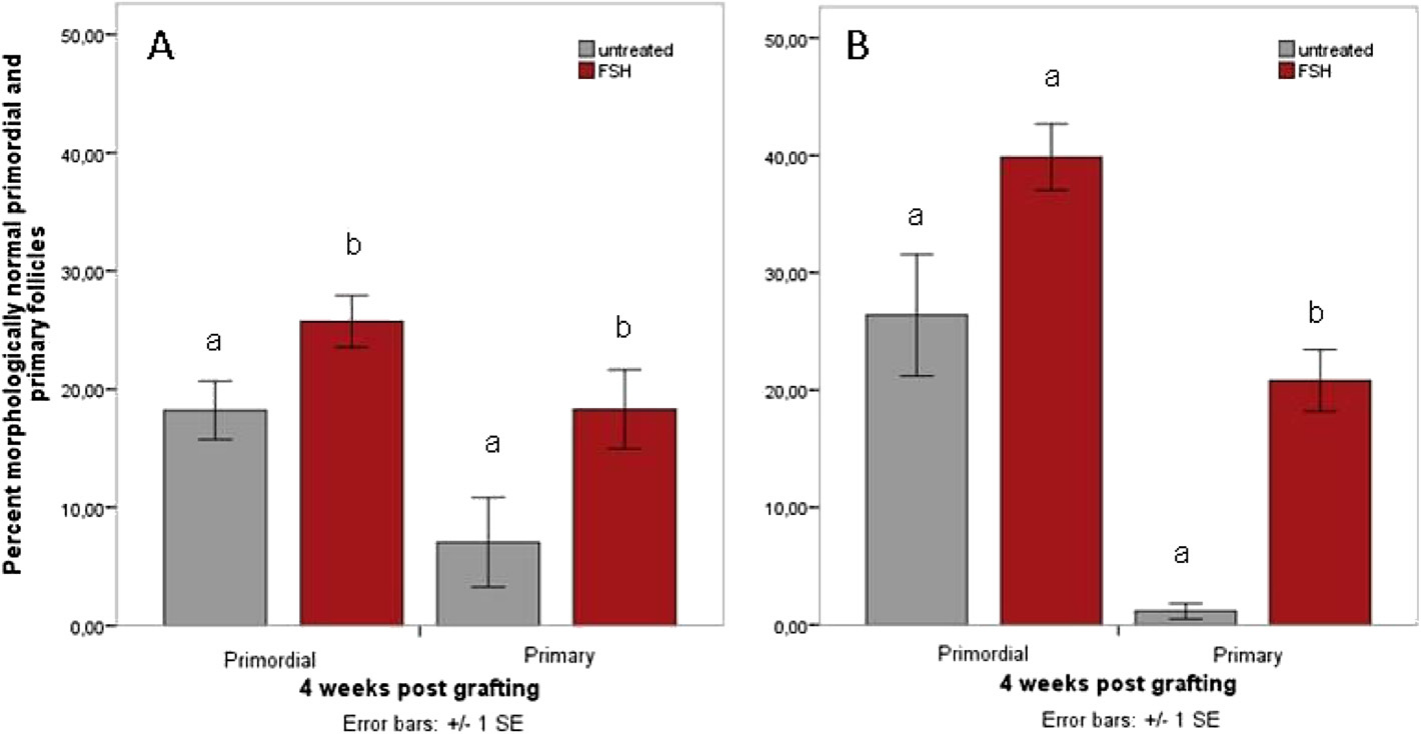
**A and B Increased normal follicular morphology following FSH treatment.** The percentage of follicles with normal morphology following grafting for up to 4 weeks in fresh (**A**) as well as frozen-thawed ovarian tissue (**B**) is higher in FSH-treated recipients.

Cryopreservation and grafting both challenge follicular morphology to a high extent. FSH treatment prior to graft explantation, however, has a protective effect on the morphology of primordial and primary follicles (Figure 5A and 5B): A significantly higher percentage of freshly grafted primordial follicles in the FSH group display normal morphological features (25.7+/−2.2%) than in the untreated group (18.2+/−2.5%, p=0.006, Figure 5A). In frozen tissues, the percentage of morphologically normal primordial follicles is also higher in the FSH group (39.8+/−2.8%) than in the untreated group (26.4+/−5.2%, Figure 5B), but this difference does not reach statistical significance (p=0.074).

Likewise, a supportive effect of FSH treatment on primary follicle morphology is found in fresh (p=0.001; Figure 5A) and frozen-thawed (p=0.000; Figure 5B) tissues and seems to be even more pronounced than in primordial follicles.

### Proportions of primary follicles in grafted tissues are influenced by FSH treatment and previous cryopreservation

**Figure 6:**
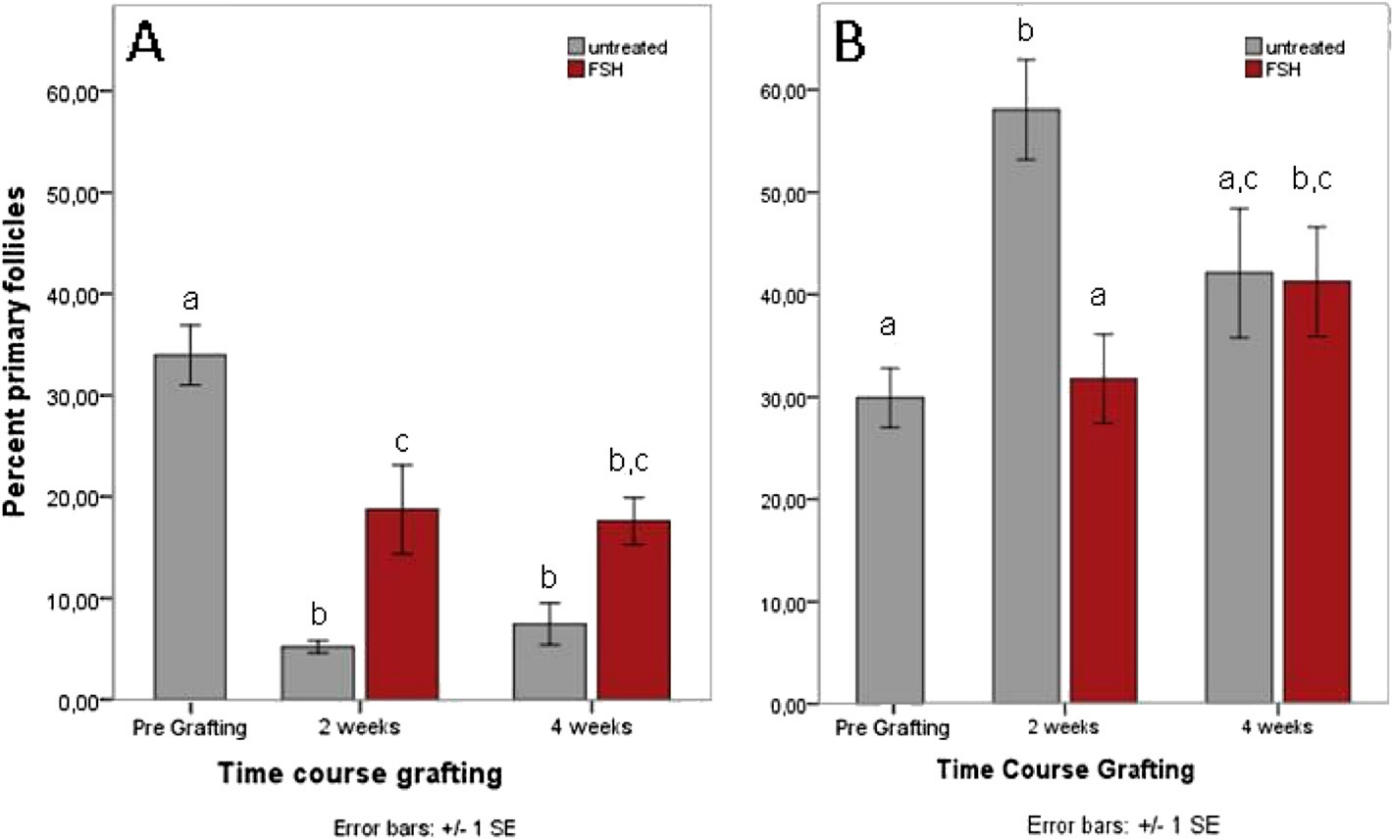
**A and B Proportions of primary follicles in grafted tissues.** The proportion of primary follicles drops to much lower values compared with the pregraft control in freshly grafted tissues (**A**), but to a smaller extent when preceded by FSH treatment. In untreated but not FSH treated tissues, the proportion of primary follicles is increased at 2 weeks compared to pregraft values in frozen-thawed ovarian tissue (**B**), and at 4 weeks this increase is significant in FSH-treated tissues only.

Changes in the proportion of primary follicles in the resting follicle pool may mirror the beginning of follicular recruitment. Before xenografting, the proportions of primary follicles are not significantly different between fresh (33.9+/−2.9%) and frozen-thawed (29.9+/−2.9%) ovarian tissues. In freshly grafted tissues, these proportions decrease significantly after transplantation and then remain stable over time (p=0.000; Figure 6A). FSH treatment prior to graft explantation substantially diminishes this effect at both 2 (p=0.015) and 4 weeks (p=0.04) post grafting (Figure 6A).

In frozen-thawed ovarian tissue, the proportion of primary follicles is significantly increased at 2 weeks after grafting in untreated (p=0.000), but not FSH-treated tissues (p=0.411; Figure 6B), reflecting a relatively smaller loss of primary compared to the dramatic loss of primordial follicles. At 4 weeks after transplantation, the proportions of primary follicles are increased in both untreated and FSH treated tissues compared to pregraft values, but largely unaffected by FSH treatment itself (p=0.458; Figure 6B).

## Discussion

The size of the resting follicle pool determines the ovarian reserve of an individual and correlates with graft sustainment after restoration of fertility by ovarian tissue cryopreservation and transplantation. Assessment of ovarian reserve following ovarian tissue transplantation is challenging when using established markers and indicates high variability between patients [21]. Besides age of the donor [22] and ongoing recruitment of follicles into the maturation pathway, it is challenged by external influences such as previous transportation [23], cryopreservation procedure and grafting conditions [24]. Xenografting to ovarectomized versus intact animals has previously been considered to be beneficial for graft survival and growth due to elevated gonadotropin levels following interruption of the inhibitory pituitary feedback loop [25,16]. Gonadotrophin treatment has been variously studied in its effect on cytoplasmic maturation and oocyte developmental competence in different species including pig [26], mouse [27] and the monkey model [28]. There are anecdotal reports on a possible effect of FSH on cyctoplasmic maturation in human oocytes [29], but the question of FSH effects on the resting follicle pool in human ovarian tissues has rarely been addressed systematically. Oktay has described FSH influence on follicular growth at earlier stages than previously suspected, although he confirmed independence of primordial follicle growth inititation from gonadotrophins [30]. The value of FSH stimulation for obtaining pregnancy in patients with autologous transplantation of ovarian tissue is still a matter of debate [31].

Enhanced follicular depletion through FSH effects during ovarian stimulation has anecdotally been reported in the human [32], but controlled experimental studies on the effect of FSH on the resting follicle pool are still missing. According to our results in the present study, initial loss of primordial and primary follicles after grafting of fresh or frozen-thawed tissues is rescued at least in parts by FSH treatment starting within the 2^nd^ week of transplantation. In freshly but not in frozen-thawed grafted tissues, a positive effect of FSH treatment on primordial follicle number was also observed if the start of treatment was postponed to 3 weeks post transplantation. As we have previously documented in the marmoset [8], and others in different species [14,33,34], cryopreservation applying a slow-freeze protocol significantly reduces normal follicular morphology in the resting follicle pool. In the present paper, we have also assessed the dramatic initial follicular loss following xenografting of frozen-thawed tissue. It is assumed that tissue damages occurring during cryopreservation and the initial avascular period after grafting are masked by a more receptive host milieu in FSH treated tissues. This is also supported by higher numbers of normal follicular morphology following FSH treatment in frozen-thawed as well as fresh tissues at all different points in time. A positive effect of FSH on follicle survival in frozen-thawed ovarian tissue applying an *in vitro* co-culture system had previously been described for other non-human primates [28]. In human ovarian tissue that was cryopreserved and xenografted to the murine back muscle, earlier beginning of vessel formation had been demonstrated following FSH stimulation [35], thus enhancing the supply with nutritional and differentiating factors. Interestingly, FSH receptor expression had been described for tumor blood vessels in various organs [36], and for ovarian surface epithelium [37], suggesting that its effect on follicular morphology is rather an indirect one and not transmitted through granulosa cells as the classical target of FSH action.

When started after a prolonged grafting period of frozen tissues, FSH negatively influences primordial follicle numbers that are already low and most likely at this point reflect manifestation of preceding tissue damages due to cryopreservation. Since primary follicle numbers are increased in these tissues, the reduction in the primordial follicle pool may additionally mirror the beginning of follicular recruitment becoming apparent in already low primordial follicle numbers. However in fresh tissues, baseline values of follicle numbers are higher also after a prolonged grafting period, and FSH treatment following early or at a later point in time after transplantation prevents primordial follicle loss. This is in agreement with viability studies on fresh and frozen ovarian tissue resulting in significantly reduced viability when applying a slow-freezing protocol [4].

Proportional analysis of primary and primordial follicle number can indicate initiation of follicular growth, and for xenografted human ovarian tissue the influence of cryopreservation [38] and timing of FSH stimulation [39] on primordial follicle activation has been speculated upon. However, in this study we have not applied any staining techniques as a definitive marker of proliferation, and higher percentages of primary follicles over all un-advanced follicles in FSH-treated freshly-grafted tissues cannot be clearly assigned to a shift from the primordial to the primary follicle compartment. From a clinical point of view this may be of outstanding importance for the efficacy of ovarian tissue grafting for fertility restoration as the majority of stored human tissues today have most likely been treated according to a slow freezing protocol, and lower chances of pregnancy from these tissues compared with fresh or vitrified tissues are disputed [10]. We have previously discussed the impact of cryopreservation *per se* on follicle pool dynamics and primary follicle activation in adult and prepubertal ovarian tissues [8]. This could rather indicate absence of inhibitory signals deriving from the stromal cells due to cryodamages than an increase in activating factors [40]. As the present study has not included in depth analysis of factors potentially involved in follicular growth and activation, involvement remains speculative. We can only assume possible secondary effects, e.g. via recruitment of antral follicles, as these were too small in number to be analyzed statistically between FSH-treated and untreated tissues. In view of an increasing clinical application of ovarian tissue grafting based on previously cryopreserved human tissues, these results may further stimulate research on intraovarian factors involved in the regulation of the resting follicle pool and positively influence clinical outcomes.

## Conclusions

FSH treatment alleviates depletion of the resting follicle pool and promotes normal follicular morphology both in freshly and frozen-thawed grafted tissues. In previously cryopreserved tissues, applying to most of the tissues intended for clinical use in fertility preservation attempts, its positive effect on primordial follicle numbers and potential graft sustainment is dependent on an early start of treatment within one week of transplantation. Whereas in fresh tissues FSH treatment following xenografting results in a higher percentage of primary over all un-advanced follicles, this appears to be largely independent of FSH in frozen-thawed tissues.

## Competing interests

All authors declare that they have no competing interests.

## Authors’ contributions

VVS and BS designed the study, analyzed and interpreted the results and drafted the manuscript. RC performed the experiments and analyzed the results. EN and LK participated in the study design, helped in the acquisition of funding and critically reviewed the manuscript. RO helped to perform statistical analysis and with the drafting of the manuscript. All authors read and approved the final manuscript.
